# PERK mediates resistance to BRAF inhibition in melanoma with impaired PTEN

**DOI:** 10.1038/s41698-021-00207-x

**Published:** 2021-07-19

**Authors:** Yifei Qin, Qiang Zuo, Lei Huang, Liping Huang, Glenn Merlino, Yanlin Yu

**Affiliations:** 1 Laboratory of Cancer Biology and Genetics, National Cancer Institute, National Institutes of Health, Bethesda, MD USA; 2 Present Address: Southern Medical University, Guangzhou, People’s Republic of China

**Keywords:** Melanoma, Targeted therapies

## Abstract

Targeting mutant BRAF in patients with melanomas harboring this oncogene has been highly successful as a first-line treatment, but other mutations may affect its efficacy and alter the route of acquired resistance resulting in recurrence and poor prognosis. As an evolving strategy, melanoma treatment needs to be expanded to include targets based on newly discovered emerging molecules and pathways. We here show that PERK plays a critical role in BRAF inhibitor-acquired resistance in melanoma with impaired PTEN. Inhibition of PERK by either shRNA or a pharmacological inhibitor blocked the growth of BRAF inhibitor-resistant melanoma with impaired PTEN in vitro and in vivo, suggesting an effective approach against melanomas with mutant BRAF and PTEN deficiency. Our current findings, along with our previous discovery that the AXL/AKT axis mediates resistance to BRAF inhibition in melanoma with wild-type PTEN, provide new insights toward a strategy for combating BRAF inhibition-acquired resistance in BRAF mutant melanoma with different PTEN statuses.

## Introduction

Cutaneous melanoma represents one of the most aggressive and difficult to treat forms of human cancer, with a worldwide incidence that has steadily increased over the past half a century^1^. It has been characterized as harboring mutations in multiple genes^2^. Oncogenic mutations in the BRAF pathway are the most well-described genetic mutations associated with melanoma development and progression^3^. More than 50% of all melanomas harbor activating BRAF kinase mutations, with BRAFV600E representing more than 90% of BRAF mutations^3,4^, the consequence of which is the constitutive activation of RAF-mitogen activated protein kinase (MAPK) and extracellular signal-regulated kinase (ERK) signaling to promote melanoma proliferation and resistance to apoptosis^5^. Nevertheless, these BRAF mutations are commonly present in benign nevi; thus, mutation of BRAF alone is not sufficient to initiate melanomagenesis^6,7^; therefore, additional oncogenic alterations are required to drive melanocyte transformation and melanoma development^8^.

The phosphatase and tensin homolog deleted on chromosome ten (PTEN) tumor suppressor is inactivated frequently in many human cancers. More than 35% of melanoma have lost PTEN function^9–11^. Inactivation of PTEN is often found in advanced melanoma and is coincident with BRAF mutation^12–14^. PTEN dephosphorylates the phosphatidylinositol (3,4,5)-trisphosphate [PtdIns (3,4,5)P3, or PIP3] and effectively antagonizes the PI3K/AKT pathway, thereby inhibiting cell proliferation and promoting apoptosis^13,15,16^. The loss of function of PTEN activates the PI3K pathway. The cooperation of oncogenic BRAF kinase mutation with inactivated tumor suppressor PTEN activates both the MAPK and PI3K pathways to promote the progression of melanoma and metastasis^17,18^. Moreover, clinical studies have reported that patients with melanoma carrying BRAF mutation and PTEN inactivation showed a trend for shorter median progression-free survival (PFS) on BRAF inhibitor-targeted therapy than patients with melanoma having wild-type PTEN^19,20^. Although recent studies have shown that melanoma cell lines with inactivated PTEN can be growth-arrested by BRAF and MEK inhibitor treatments, they are resistant to apoptosis induction^21^. These studies suggested that mutant PTEN causes an inadequate response to current anti-mutant BRAF kinase treatments in melanoma, supporting the notion that PTEN inactivation identifies a distinct clinically significant subset of melanomas, while implying that PTEN status may affect the molecular mechanism of late acquired resistance to BRAF inhibitor (BRAFi). A recent study has shown that the loss of PTEN contributes to intrinsic resistance to BRAFi via the suppression of BIM-mediated apoptosis^22^. Knock-down of PTEN conferred vemurafenib resistance, while re-expression of PTEN conferred vemurafenib sensitivity^23,24^. However, most BRAF-mutant melanomas with PTEN inactivation appear to be sensitive to BRAF inhibition^25^, indicating that the required resistance mechanism associated with PTEN mutation is complex and remains unclear. We hypothesized that inactivated PTEN alters downstream pathways to contribute to acquired BRAFi resistance in melanoma.

To understand the molecular mechanism of PTEN in resistance to BRAF inhibition, we previously created BRAFi-resistant melanoma models with/without wild-type PTEN. We found that the hyperactivation of both ERK and AKT pathways was associated with BRAFi resistance in melanoma with wild-type PTEN^26^. PTEN-inactivated melanoma cells required only the ERK resistance mechanism. Moreover, we identified AXL as a critical upstream effector of AKT pathway-associated resistance to BRAFi in melanoma with wild-type PTEN^26,27^. In this study, we determined that PERK (protein kinase RNA-like endoplasmic reticulum kinase, or EIF2AK3), an ER stress sensor, is an essential mediator associated with resistance to BRAFi in melanoma with inactivated PTEN. Moreover, using knockout PTEN models by gene editing approaches, we confirmed that PERK-mediated resistance only happened in melanoma with PTEN deficiency. Notably, we demonstrated that the inhibition of PERK by shRNAs or small molecular inhibitors enhanced the sensitivity of resistant PTEN-inactivated melanoma cells to BRAFi and blocked their growth in vitro and in vivo. These findings have uncovered the mechanism by which PTEN inactivation contributes to acquired resistance to BRAFi and offer a rational strategy to guide clinical testing in subsets of patients who relapse during treatment with BRAFi.

## Results

### PERK is upregulated in BRAFi-resistant human melanoma with impaired PTEN

**Fig. 1:**
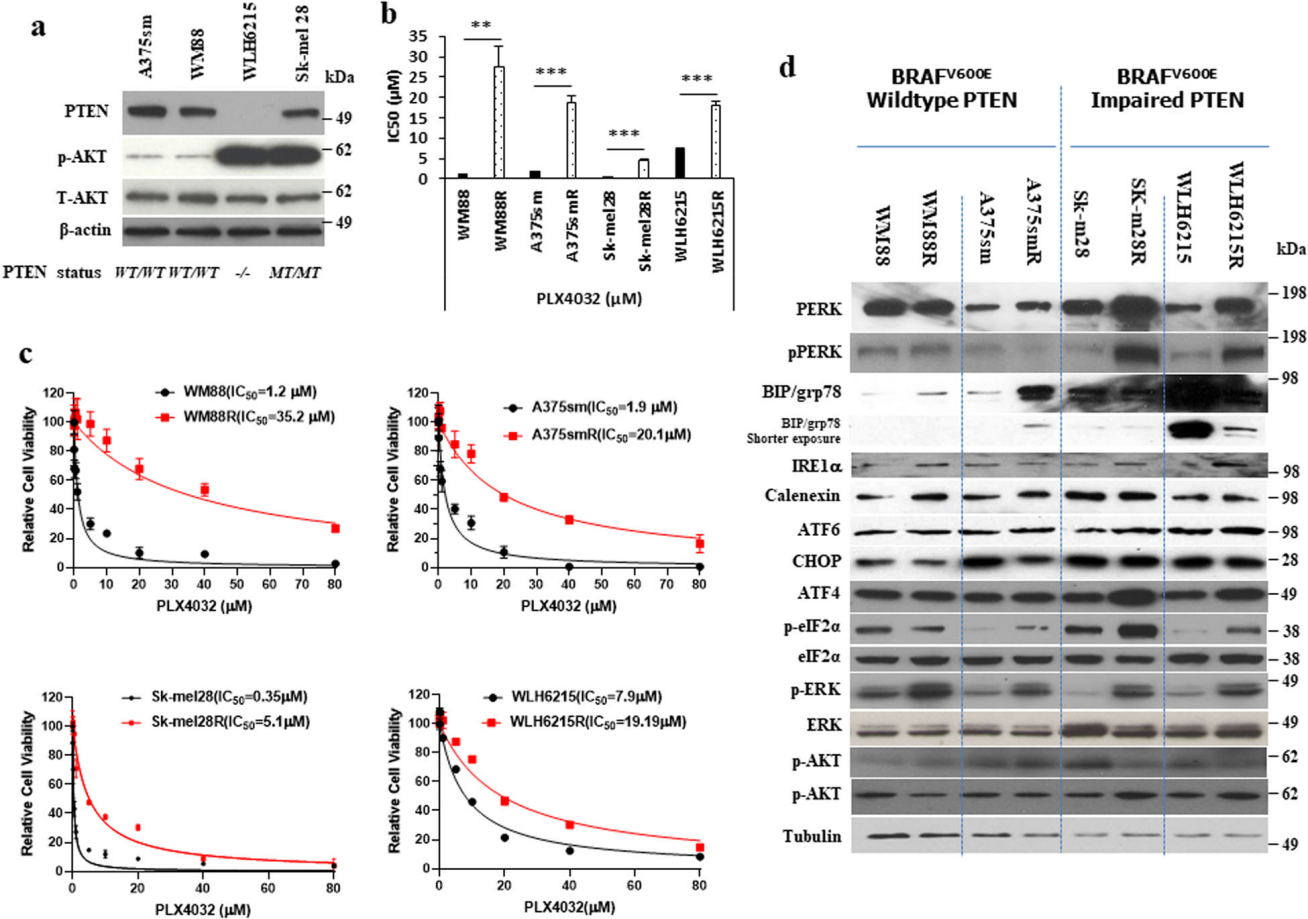
PERK was upregulated in BRAFi-resistant human melanoma cells with impaired PTEN. **a** The western blotting showed the protein level of PTEN, pAKT, and AKT in BRAF^V600E^ human melanoma cell lines with various PTEN genetic backgrounds. The protein level of β-actin is the loading control. **b** The values of 50%-inhibitory concentration (IC50) of 4 pairs of parental and resistant human melanoma cells were determined using CCK8 assays. Error bars denote s.d. for biological three repeats. Results are statistically significant between parental and resistant groups by Student’s *t*-test (*p < 0.05, **p < 0.01, ***p < 0.001). **c** The cell viability curves of 4 pairs of parental (WM88, A375sm, Sk-mel28, and WLH6215) and resistant (WM88R, A375smR, Sk-mel28R, and WLH6215R) human melanoma cells were determined using CCK8 assays. Error bars denote s.d. for biological three repeats. **d** The western blotting showed the indicated protein level in BRAF inhibitor-resistant melanoma cell lines with various PTEN genetic backgrounds, the protein level of tubulin as the loading control.

To examine the role of PTEN in resistance to BRAF inhibition, we created BRAFi-resistant melanoma models with/without wild-type PTEN. We previously found that ERK signaling was hyper-reactivated in all BRAFi-resistant melanoma cells as a major pathway of acquired BRAFi resistance independent of PTEN status. However, the PI3K/AKT signaling pathway was hyperactivated only in BRAFi-resistant melanoma cells with wild-type PTEN (Fig. 1d). We then identified that AXL is a key upstream effector of AKT pathway-associated resistance to BRAFi in melanoma with wild-type PTEN^26^, but not in melanoma with PTEN loss or inactivated PTEN^26,27^, implicating the existence of a different mechanism of BRAFi resistance in melanoma with impaired PTEN.

Oncogenic BRAF induces chronic ER stress conditions resulting in increased basal autophagy and apoptotic resistance of cutaneous melanoma^28^. Studies have shown that targeting oncogenic BRAF kinase by BRAF inhibiter could also activate ER stress signaling pathways^29,30^. Inactivation of PTEN would relieve ER stress and enhance cell survival^31^.

To evaluate whether the ER stress pathway is involved in the regulation of BRAFi resistance in melanoma, we examined the expression of ER stress proteins within our four BRAFi-resistant melanoma models [parental and resistant (labeled with R)], including two melanomas with wild-type PTEN (WM88 and WM88R; A375sm and A375smR) and two melanomas with impaired PTEN (WLH6215 and WLH6215R, total loss PTEN gene; Sk-mel28 and Sk-mel28R, the lost function of PTEN via phosphatase mutation), which loss the phosphatase activity of PTEN (Supplementary Fig. 1a) with hyperactivated AKT (Fig. 1a and Supplementary Fig. 5). Consistent with our previous study^26^, all BRAFi-resistant cells presented the resistance to not only PLX4032 (Fig. 1b, c) but also to other BRAFi PLX4720, Babrafenib, and MEK inhibitor (MEKi) Trametinib (Supplementary Fig. 1b–g). Interestingly, the BRAFi-resistant melanoma with wild-type PTEN has high potential resistance to MEK inhibitor Trametinib with more than 50 higher resistance indexes (RI) [WM88 (IC50 = 0.031 µM)/WM88R (IC50 = 38.87 µM, RI = 1253.8; A375sm (IC50 = 11.38 µM)/A375smR (791.6 µM), RI = 69.56)]. Both BRAFi-resistant melanoma cell lines with wild-type PTEN do not become sensitive to MEK inhibitor. Although the BRAFi-resistant melanoma with impaired PTEN also has the ability of resistance to MEKi, their IC50s are less than 1.2 µM [Sk-mel28(IC50 = 0.015 µM)/Sk-mel28R (IC50 = 0.14 µM), RI = 9.3; WLH2615 (IC50 = 0.56 µM)/WLH6215R(IC50 = 1.2 µM), RI = 2.1]. These BRAFi-resistant melanomas with impaired PTEN are still sensitive to MEKi (Supplementary Fig. 1b–g). We found that ER proteins IRE1α, Atf6, Calnexin, and CHOP are all equally expressed in resistant melanoma cells (Fig. 1d and Supplementary Fig. 5). Bip (GRP78) was found to increase in BRAFi-resistant melanoma with wild-type PTEN but did not change or even decreased in melanoma with impaired PTEN (Fig. 1d). Interestingly, PERK increased in BRAFi-resistant melanoma with impaired PTEN, not in resistant melanoma with wild-type PTEN (Fig. 1d). Moreover, we also found that ATF4 and phosphorylated eIF2α upregulated in PTEN mutant BRAFi-resistant melanoma (Fig. 1d). eIF2α and ATF4 are downstream of the PERK signaling pathway, suggesting PERK signaling may play the role of BRAFi resistance. PERK is one of the most well-known ER stress sensors for initiating “unfolded protein response” (UPR) signaling^32,33^. Several studies have shown that PERK signaling is associated with multi-drug resistance in breast cancer^34^ and with imatinib (BCR-ABL inhibitor) resistance in chronic myeloid leukemia (CML)^35^. Together with these, increased PERK may also contribute to resistance to BRAFi in melanoma with impaired PTEN.

### A PERK inhibitor can inhibit the cell survival of BRAFi–resistant melanoma with impaired PTEN

**Fig. 2:**
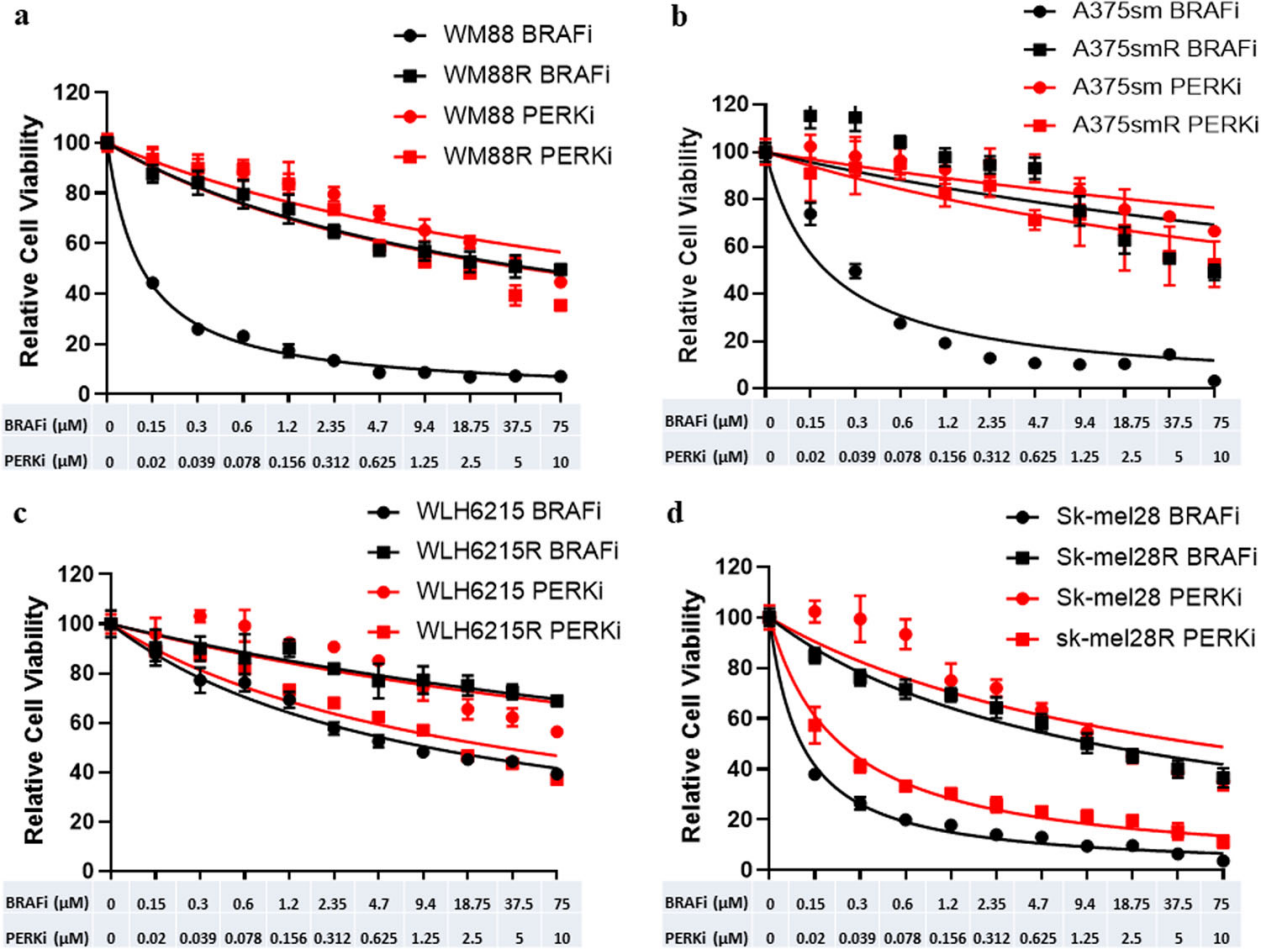
A PERK inhibitor could inhibit the cell survival of BRAFi–resistant melanoma with impaired PTEN. WM88 (**a**) and A375sm (**b**) cells with wild-type PTEN or WLH6215 (**c**) and Sk-mel28 (**d**) cells with impaired PTEN and their PLX4032-resistant counterparts were treated with different concentrations of PLX4032 BRAF inhibitor (BRAFi) and GSK2606414 PERK inhibitor (PERKi). The cell viability curves of parental and resistant human melanoma cells were determined using CCK8 assays. Error bars denote s.d. for biological three repeats.

To evaluate whether PERK is involved in the regulation of resistance to BRAFi in melanoma, we first tested the ability of a PERK inhibitor GSK2606414 (PERKi)^36^ to block cell proliferation (viability) of BRAFi-resistant melanoma compared with the treatment of BRAFi. As Fig. 2 shows, the BRAFi inhibits the cell growth of all parental melanoma cells. In contrast, our BRAFi-resistant melanoma cell lines resist the inhibition of BRAFi, as expected (Fig. 2). Interestingly, all melanomas with wild-type PTEN, both parental and resistant cells, grew with a similar rate under treatment with different doses of the PERKi (Fig. 2a, b). In contrast, the PERKi could significantly inhibit the growth of both BRAFi-resistant melanoma cell lines with impaired PTEN, while exhibiting little effect on the growth of both their parental melanoma cell lines (Fig. 2c, d). Moreover, the combination with BRAFi, the PERKi did not significantly affect the ability of BRAFi in treatment for the BRAFi-resistant melanoma with wild-type PTEN(IC50 of BRAFi without/with PERKi in WM88R and A375smR were 6.8 µM/4.1 µM and 14.3 µM/9.6 µM, respectively), in contrast, the combination with PERKi could significantly low the IC50 of BRAFi in BRAFi-resistant melanoma with impaired PTEN (IC50 of BRAFi without/with PERKi in Sk-mel28R and WLH6215R were 5.07 µM/0.93 µM and 16.5 µM/3.9 µM, respectively), suggesting that PERKi enhanced the sensitivity (Supplementary Fig. 2) of BRAFi with synergistic effect (combination index CI < 1) (Supplementary Figs. 3 and 4) in the BRAFi-resistant melanoma with impaired PTEN. Together, the data indicate that PERK may help regulate resistance to BRAF inhibition in melanoma with impaired PTEN.

### PERK is required for acquired resistance to BRAFi in melanoma with impaired PTEN

**Fig. 3:**
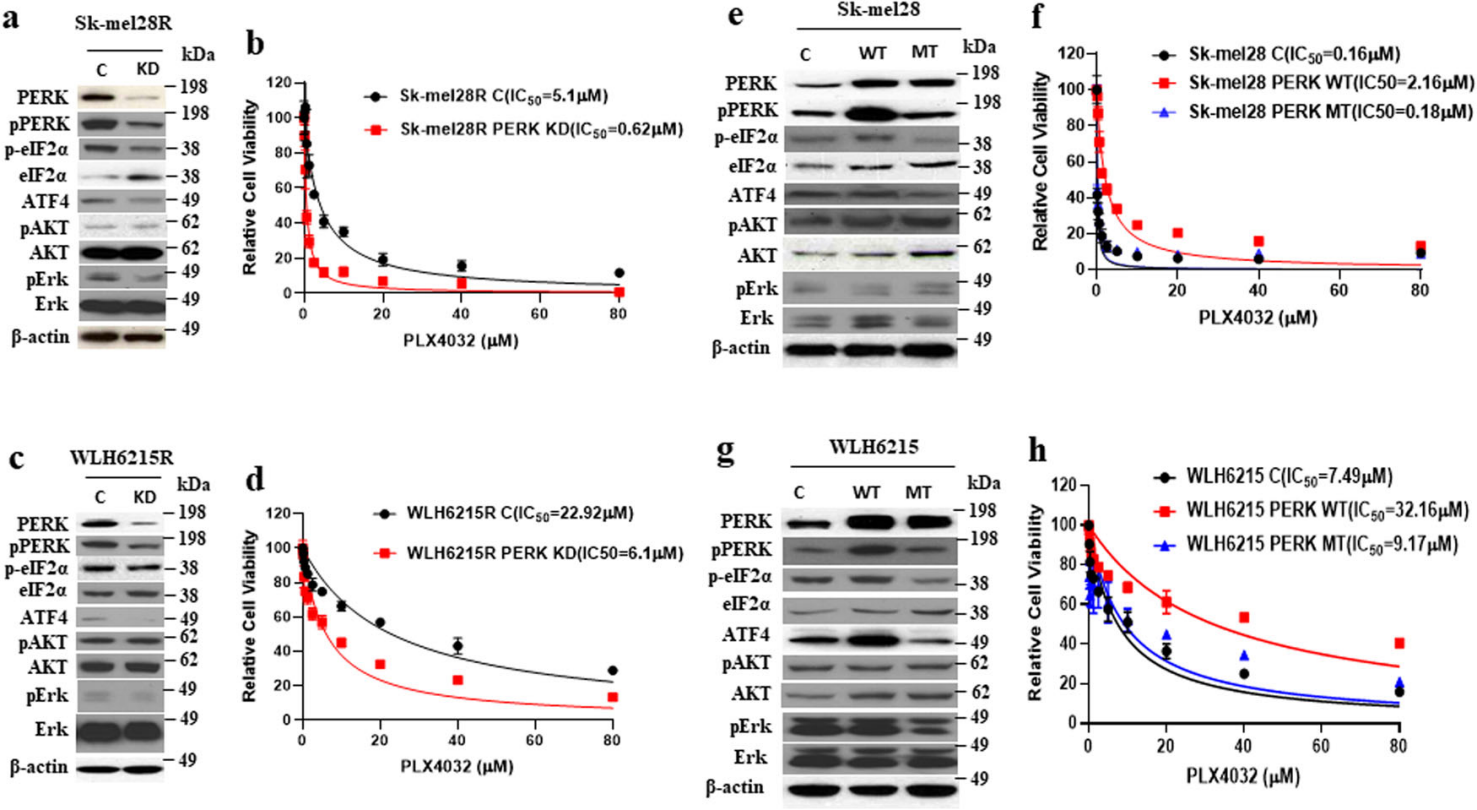
PERK affected the ability of resistance to BRAF inhibitors in melanoma with impaired PTEN. **a**, **b** and **c**, **d** Knock-down of PERK enhances the sensitivity to BRAFi in PTEN impaired melanoma. **a**, **c** Western blotting showed the indicated protein level in PERK knockout cells from Sk-mel28R (**a**) and WLH6215R (**c**). C, empty vector; KD, PERK knock-down. **b**, **d** BRAFi-resistant Sk-mel28R (Sk-mel28R C) and its PERK knock-down (Sk-mel-28R PERK KD) or WLH6215R (WLH6215R C) and its PERK knock-down (WLH6215R PERK KD) cells were treated with different concentrations of BRAF inhibitor PLX4032. The cell viability curves of Sk-mel28R (Sk-mel28R C) and its PERK knock-down (Sk-mel-28RPERK KD) (**b**) or WLH6215R (WLH6215R C) and its PERK knock-down (WLH6215R PERK KD) (**d**) human melanoma cells were determined using CCK8 assays. Error bars denote s.d. for biological three repeats. **e**, **f** and **g**, **h** Overexpression of PERK can increase the ability of resistance to BRAFi in PTEN impaired melanoma. **e**, **g** Western blotting showed the indicated protein level in PERK overexpressing cells from Sk-mel28 (**e**) and WLH6215 (**g**). C, empty vector; WT, wild-type PERK; MT, mutant PERK. **f**, **h** Cell viability curves of Sk-mel28 (Sk-mel28 C) and its overexpressing (Sk-mel28 PERK WT and Sk-mel28 PERK MT) (**f**) or WLH6215 (WLH2615 C), and it’s overexpressing (WLH2615 PERK WT and WLH2615 PERK MT) (**h**) cells in response to PLX4032 were determined using CCK8 assay. Error bars denote s.d. for biological three repeats.

To confirm the function of PERK in the regulation of the resistance to BRAFi in BRAF^V600E^ mutant melanoma with impaired PTEN, we examined whether downregulation of PERK in BRAFi-resistant, PTEN-deficient melanoma with elevated PERK could rescue the sensitivity of resistant melanoma to BRAF inhibition. We knocked down the protein level of PERK in the WLH6215R and Sk-mel28R melanoma cells by shRNA. Knock-down of PERK downregulated the ATF4 and phosphorylated eIF2α in both cell lines (Fig. 3a, c and Supplementary Fig. 5). Interestingly, knock-down of PERK in both resistant melanoma cell lines with impaired PTEN enhanced the sensitivity to BRAF inhibitor (Fig. 3b, d), indicating that PERK is involved in regulating resistance to BRAF inhibition in PTEN impaired melanoma. To further confirm the importance of PERK in resistance to BRAF inhibition in BRAF^V600E^ mutant melanoma with impaired PTEN, we introduced a wild-type form and a function lost mutant form of PERK into both parental BRAF^V600E^-mutant melanoma cells Sk-mel28 and WLH6215 and tested resistance to BRAF inhibition. As expected, transfected cells with WT PERK expressed a higher level of PERK and p-PERK as well as increased eIf2α and ATF4, while PERK mutant form did not affect the phosphorylation of PERK and eIF2α as well as ATF4 (Fig. 3e, g and Supplementary Fig. 5). Notably, overexpression of wild-type PERK could increase the capacity of resistance to BRAFi in both parental BRAF^V600E^-mutant melanoma Sk-mel28 and WLH6215 with impaired PTEN, but the mutant form of PERK did not (Fig. 3f, h), indicating overexpression of PERK confers BRAFi resistance in melanoma. Altogether, our data demonstrated that PERK is involved in the regulation of resistance in PTEN impaired melanoma.

**Fig. 4:**
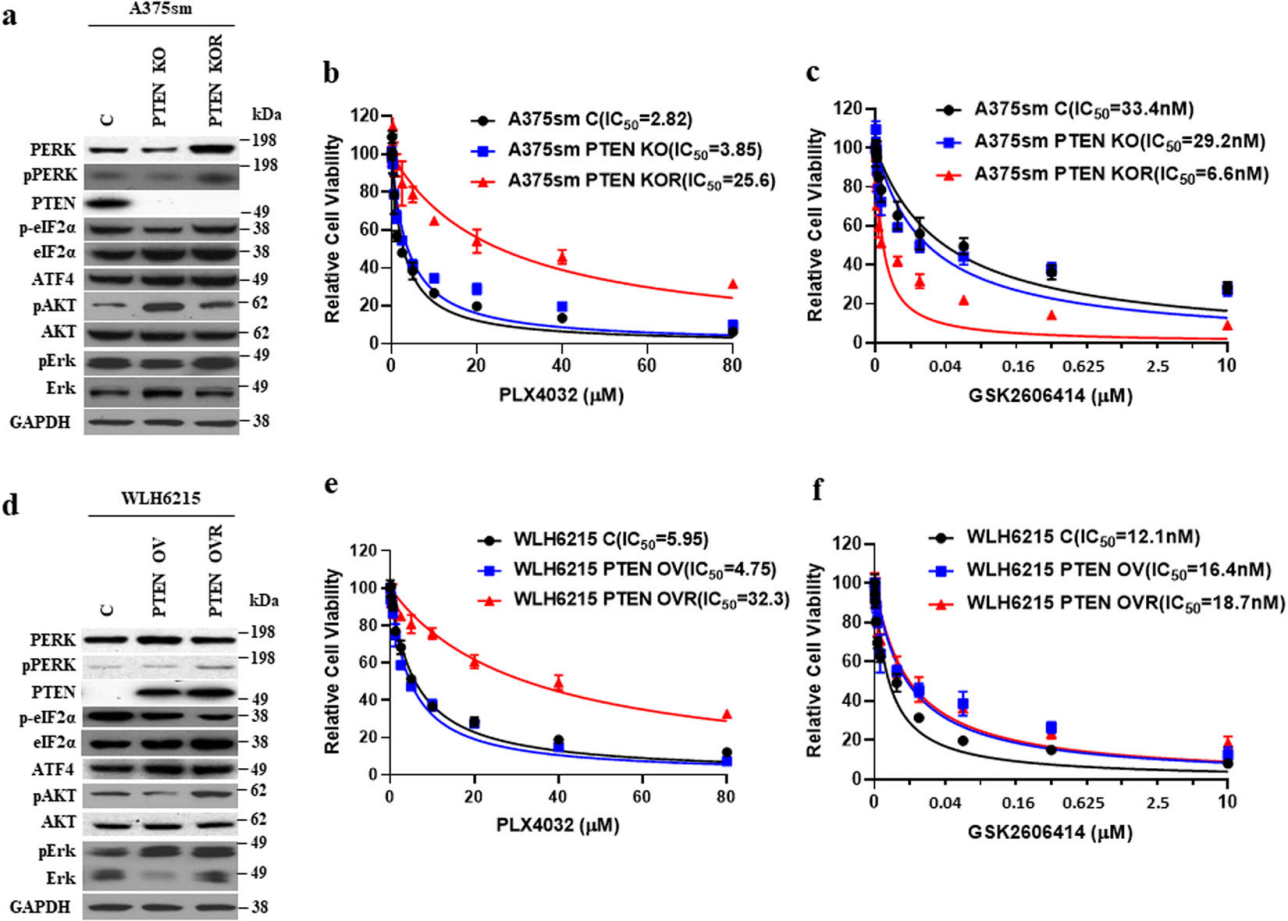
PTEN status influences the expression of PERK in BRAF ^V600E^-mutated-resistant melanoma. **a** Western blot showed the indicated protein level in A375sm. C, empty vector; PTEN KO, PTEN knockout by gene editing; PTEN KOR, PTEN knockout A375sm resistance to BRAF inhibition. **b** Cell viability curves of A375sm with empty vector (A375sm C), A375sm PTEN knockout (A375sm-PTEN KO), and its BRAF inhibitor-resistant counterpart A375sm PTEN KO-R (A375sm-PTEN KOR) cells in response to PLX4032 were determined using CCK8 assay. Error bars denote s.d. for biological three repeats. **c** Cell viability curves of A375sm with empty vector (A375sm C), A375sm PTEN knockout (A375sm-PTEN KO), and its BRAF inhibitor-resistant counterpart A375sm PTEN KO-R (A375sm-PTEN KOR) cells in response to PERK inhibitor GSK2606414 were determined using CCK8 assay. Error bars denote s.d. for biological three repeats. **d** Western blot showed the indicated protein level in WLH6215 transfected with empty vector (C) and PTEN. C, empty vector; PTEN OV, PTEN overexpressing; PTEN OVR, PTEN overexpressing WLH6215 resistance to BRAF inhibition. **e** Cell viability curves of WLH6215 with empty vector (WLH6215 C), WLH6215 PTEN overexpressing (WLH6215 PTEN OV), and its BRAF inhibitor-resistant counterpart WLH6215 PTEN OV-R (WLH6215 PTEN OVR) cells in response to PLX4032 were determined using CCK8 assay. Error bars denote s.d. for biological three repeats. **f** Cell viability curves of WLH6215 with empty vector (WLH6215 C), WLH6215 PTEN OV (WLH6215 PTEN OV), and its BRAF inhibitor-resistant counterpart WLH6215 PTEN OVR (WLH6215 PTEN OVR) cells in response to PERK inhibitor GSK2606414 were determined using CCK8 assay. Error bars denote s.d. for biological three repeats.

To confirm that the regulation of resistance by PERK is dependent on PTEN status in melanoma, we examined the effects of inhibitor treatments and PERK expression on cell viability in our other two pairs of parental and BRAFi-resistant melanoma cell lines. These are A375sm PTEN KO/A375sm PTEN KO-R (parent and resistant A375sm cells with PTEN knockout) (Fig. 4a and Supplementary Fig. 5)^26^ and WLH6215 PTEN/WLH6215 PTEN-R (parent and resistant WLH6215 cells ectopically expressing wild-type PTEN) (Fig. 4d). Consistent with our previous study^26^, both A375sm PTEN KO-R and WLH6215 PTEN-R have the ability to resist the BRAFi PLX4032 (Fig. 4b, e). Interestingly, upon examination of PERK expression, we found that A375sm PTEN KO-R expressed a higher level of PERK and phosphorylated PERK (Fig. 4a) and was sensitized to PERK inhibition (Fig. 4c), implying that the inactivation of PTEN in melanoma may alter the path to resistance to BRAF inhibition and induce expression of PERK. Moreover, WLH6215 PTEN-R cells, which re-express the wild-type PTEN and become resistant to BRAF inhibitor, have a similar level of PERK with WLH6215 PTEN and WLH6215 and were not sensitized to PERK inhibition (Fig. 4f). As with WM88R and A375smR melanoma cells with wild-type PTEN, the level of PERK in resistant cells did not change (Fig. 4d and Supplementary Fig. 5). These data confirmed that regulation of resistance by PERK is dependent on PTEN status in melanoma.

### A PERK pharmacological inhibitor induces apoptosis and inhibits the growth of BRAF mutant-resistant PTEN-deficient melanoma in vivo

**Fig. 5:**
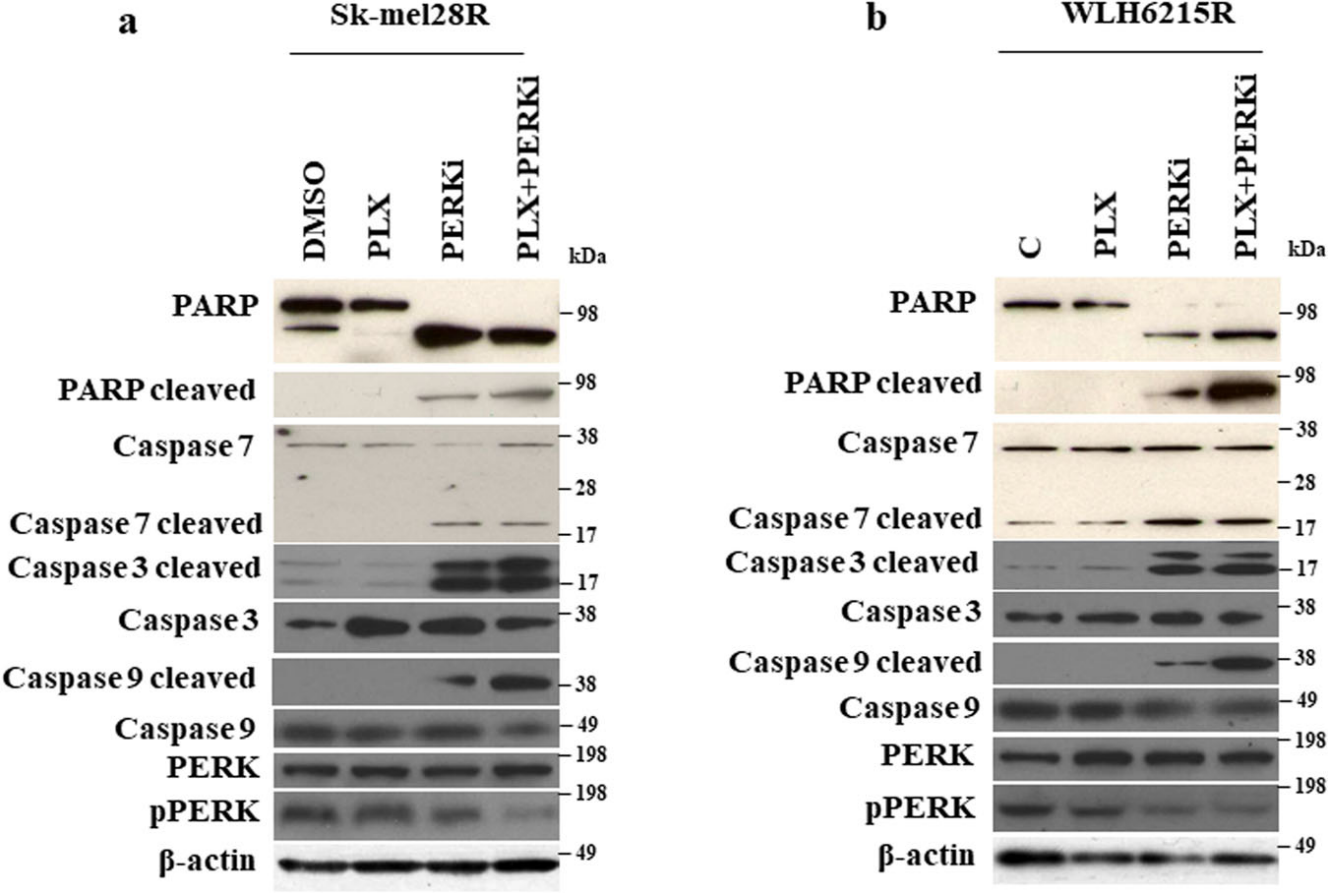
Inhibition of PERK by a small molecular inhibitor induced tumor cell apoptosis. **a**, **b** Western blot showed the indicated protein levels in human BRAFi-resistant melanoma Sk-mel28R (**a**) and WLH6215R (**b**) treated with 5 μM of BRAFi PLX4032 or 5 nM of PERK inhibitor GSK2606414 and combination of BRAFi and PERKi for 48 h. DMSO, as control; PLX, BRAF inhibitor PLX4032; PERKi, PERK inhibitor GSK2606414; PLX + PERKi, the combination of BRAFi PLX and PERKi GSK2606414. The protein of β-actin as a loading control.

To examine the mechanisms underlying the observed responses, we treated the BRAFi-resistant PTEN impaired melanoma cells with BRAF and PERK inhibitors and checked the expression of apoptotic markers (Fig. 5). Consistent with previous studies^34,36^, the PERK inhibitor (PERKi) GSK2606414 could inhibit the activity of PERK in both BRAFi-resistant melanoma cells (Fig. 5). Moreover, we found that treatment with the PERK inhibitor GSK2606414 alone or in combination with BRAF inhibitor PLX4032 increased the protein level of cleaved PARP, cleaved caspase-7, cleaved caspase-3, and cleaved caspase-9 in BRAFi-resistant melanoma cells (Fig. 5 and Supplementary Fig. 5), suggesting that the inhibition of PERK by a small molecular inhibitor could promote tumor cell apoptosis.

**Fig. 6:**
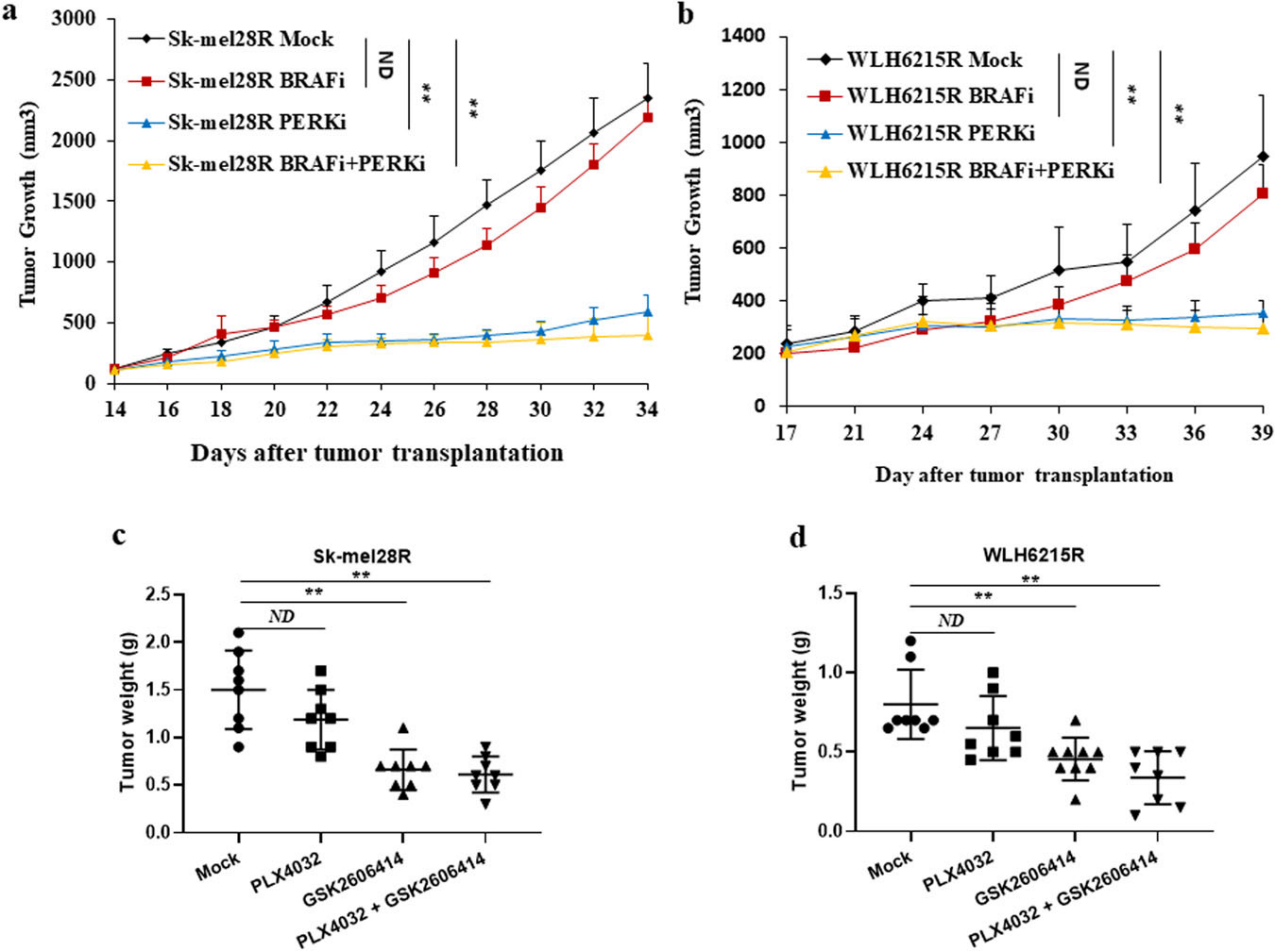
Inhibition of PERK by a pharmacological inhibitor GSK2606414 blocked tumor growth in vivo. **a** The growth curves of the resistant Sk-mel28R tumor cells treated with mock (Sk-mel28R Mock, *n* = 8), BRAF inhibitor PLX4032 (Sk-mel28R BRAFi, *n* = 8), PERK inhibitor GSK2606414 (Sk-mel28R PERKi, *n* = 8), or a combination of BRAF inhibitor PLX4032 with PERK inhibitor GSK2606414 (Sk-mel28R BRAFi + PREKi, *n* = 8). **b** The growth curves of WLH6215R tumor cells treated with mock (WLH6215R Mock, *n* = 8), BRAF inhibitor PLX4032 (WLH6215R BRAFi, *n* = 8), PERK inhibitor GSK2606414 (WLH6215R PERKi, *n* = 9), or combination of BRAF inhibitor PLX4032 with PERK inhibitor GSK2606414 (WLH6215R BRAFi + PREKi, *n* = 8). Each point represents mean tumor volume ± s.e.m. ND, no statistical difference; *p < 0.05; **p < 0.01; ***p < 0.001. **c**, **d** The tumor weight (g) of resistant Sk-mel28R (**c**) and WLH6215R (**d**) tumor cells treated with mock (Mock), BRAF inhibitor (PLX 4032), PERK inhibitor (GSK2606414), or a combination of PERK inhibitor GSK2606414 with BRAF inhibitor PLX4032 (PLX4032 + GSK2606414). ND, no statistical difference; *p < 0.05; **p < 0.01; ***p < 0.001.

To validate our findings for potential therapeutic utility, we designed and performed in vivo studies using xenograft preclinical mouse models (Fig. 6). PLX4032 and GSK2606414^36^ were administered to athymic nude mice orally once daily for 20–22 days starting 14- or 17-days after transplantation of the BRAFi-resistant melanoma cells with impaired PTEN, Sk-mel28R, or WLH6215R, by subcutaneous injection.

As expected, the tumors arising from resistant Sk-mel28R and WLH6215R cells treated with PLX4032 grew similarly to those treated with vehicle control (Fig. 6a, b). However, the PERK inhibitor GSK2606414 or a combination of GSK2606414 with PLX4032 significantly inhibited Sk-mel28R and WLH6215R melanoma growth in mice compared with their vehicle control (Fig. 6a, b) and reduced the weight of resistant Sk-mel28R and WLH6215R melanomas significantly (Fig. 6c, d). Our data demonstrate that blocking PERK activation inhibits BRAFi-resistant melanoma growth and suggest that PERK is a promising diagnostic marker and therapeutic target for BRAFi-resistant melanoma with impaired PTEN.

## Discussion

Malignant melanoma is characterized by a high intra-tumor and inter-tumor molecular heterogeneity with a broad spectrum of mutations in multiple genes and an inadequate response to current therapy^37,38^. For example, although the majority of melanomas carry one or more BRAF mutations, many studies have demonstrated that BRAF mutations are sufficient for the development of early lesion, but additional genetic alterations are needed for malignant transformation^6–8,39–41^. The loss of function of tumor suppressor PTEN is often found in the late stage of malignant melanoma and coincident with BRAF mutations^39–41^. The inactivated PTEN loses the ability to regulate PI3K and AKT, resulting in the hyperactivation of the PI3K/AKT pathway, promoting melanoma proliferation, migration, and metastasis^26,42^. The coexistence of BRAF mutations and PTEN inactivation does not only promote melanogenesis but also alters the response to current targeted therapy in melanoma, leading to drug resistance^26,27^. Our previous study^26^ selected BRAF^V600E^ mutant melanoma cells with wild-type or impaired PTEN and established BRAF inhibitor-resistant melanoma models (wild-type PTEN/impaired PTEN). By comparing the BRAF inhibitor-resistant melanoma with wild-type and impaired PTEN, we found that the hyperactivation of both ERK and AKT pathways was associated with BRAFi resistance in melanoma with wild-type PTEN and identified the critical role of the AXL/AKT axis in mediating the resistance to BRAFi in BRAF^V600E^ mutant melanoma with wild-type PTEN^26,27^. In this study, we demonstrate that PERK, protein kinase RNA-like endoplasmic reticulum kinase, is an essential mediator related to resistance to BRAFi in melanoma with impaired PTEN.

PERK is an endoplasmic reticulum (ER) stress sensor. Under stressful conditions, the cell alters protein homeostasis, resulting in the accumulation of unfolded proteins to activate the unfolded protein response (UPR). This response includes the release of the binding immunoglobulin protein (BiP) [also known as glucose-regulated protein 78 kDa (GRP78)] from binding to PERK, IRE1α, and/or ATF6 and subsequent binding to an unfolded protein, thereby activating these three UPR sensors (PERK, IRE1α, and ATF6), triggering either cell death or restoring protein homeostasis^32,33^. Recently, accumulating data showed that the progress of tumorigenesis causes chronic ER stress and that UPR can induce tumor proliferation^43^. The activation of the UPR in cancer cells enables their adaption to such ER stress, resulting in apoptosis resistance through the persistent expression of pro-survival instead of pro-apoptotic proteins. The UPR may serve a pro-tumorigenic role by increasing the protein folding capacity and prolonging resistance to anti-cancer inhibition^44^. Many studies have reported that PERK also mediates tumor progression and drug resistance^45^. For example, the activation of PERK promotes tumor metastasis by induction of cell epithelial to mesenchymal transition (EMT)^46^. In melanoma, BRAF mutation activates PERK and ATF4 to enhance the survival of melanoma^28^. Similar to our finding, Ma et al. reported that treatment with BRAF inhibitor induces the translocation of mutant BRAF to the ER and enhances BiP/GRP78 binding to mutant BRAF, sequestrating the release and activation of PERK and mediating melanoma drug resistance^30^. Several other studies have also shown that the PERK/elF2α/ATF4 axis promotes therapy resistance in hypoxia tumor cells^47,48^. In fact, blocking BRAF and PERK induces apoptosis in resistant cell lines^49,50^.

As reported, oncogenic BRAF mutation induces chronic ER stress conditions resulting in increased basal autophagy and senescence bypass, promoting the resistance to apoptosis in melanoma^51^. Also, the inhibition of the BRAF kinase upregulates multiple markers of the ER stress response and triggers the association of BRAF^V600E^ with the ER chaperone GRP78, which correlates with dissociation of GRP78 from the known ER stress response activator PERK. BRAF inhibition stimulates PERK activation, and knock-down or inhibition of PERK reduces BRAFi-induced autophagy and enhances apoptosis, indicating that PERK-dependent ER stress activates cytoprotective autophagy in response to BRAF inhibition^30,52^. Significantly, in concordance with these findings, combined inhibition of BRAF^V600E^ and autophagy induced a considerable regression of BRAFi-resistant melanoma in vivo^30^.

By comparing ER stress-related protein expression, we noticed that PERK was the only gene significantly upregulated in both BRAFi-resistant melanoma cells with impaired PTEN but not in BRAFi-resistant melanoma with wild-type PTEN. Interestingly, we found that BiP was decreased in BRAFi-resistant melanoma cells with impaired PTEN; this reduced BiP allowed more PERK release and activation. This analysis led us to investigate whether the activation of PERK was responsible for the resistance to BRAF inhibition. Our data showed that forced expression of PERK conferred resistance to BRAF inhibitor in the parental cell with inactivated PTEN human melanoma cells. In contrast, the knock-down of PERK reversed the resistant phenotype of resistant melanoma with inactivated PTEN.

It is noteworthy that PERK upregulation associating with BRAFi resistance in melanoma is dependent on the PTEN status in our models. When the original melanoma cells with wild-type PTEN were changed to inactivated PTEN status, they became resistant to BRAF inhibition; they had similarly upregulated PERK, confirming that PERK upregulation in resistant melanoma is dependent on PTEN. Our data support the notion that loss of PTEN alters molecular pathways to promote melanoma therapeutic resistance^26^. In fact, many studies showed that the loss of tumor suppressor PTEN leads to tumor progression and metastasis, as well as therapeutic resistance in many cancer^31,53,54^. An analysis of 66 patients treated with BRAF inhibitor by Catalanotti and colleagues showed that loss of function of PTEN is significantly associated with resistance to BRAF inhibition in metastatic melanoma^55^. Indeed, PTEN loss promotes BRAF inhibitor resistance to melanoma by inhibiting pro-apoptotic gene Bim expression^22^. In contrast, expressed PTEN regulates IGF1R-mediated BRAF inhibitor resistance in some melanoma, while melanoma with PTEN loss decreased IGF1R^56^. In our previous study, we found that the AXL/AKT pathway promotes BRAF inhibitor resistance in melanoma with wild-type PTEN, but not in melanoma with impaired PTEN^26^, indicating that PTEN status affects the molecular route to resistance to BRAF inhibition in melanoma^27^.

Recent studies have shown that tumor growth is caused by a transcriptional response through the elf2α-kinase PERK and ATF4, which activate the expression of metabolic enzymes, nutrient transporters, and mitochondrial chaperones. Alasiri et al. reported that PERK (eIF2AK3) is a direct downstream transcriptional target of FOXO3 in drug-resistant breast cancer cells^57^. They showed that these drug-resistant cells are specifically sensitive to PERK inhibition, revealing a chemotherapeutic drug-resistant cancer cell vulnerability in PERK and suggesting PERK as a potential target for cancer therapy, specifically in the context of drug-resistant cancers^58^.

We established a unique BRAFi-resistant melanoma models resistance to not only PLX4032 but also to other BRAFi PLX4720, Babrafenib, and MEK inhibitor (MEKi) Trametinib. We identified that PERK plays a critical role in BRAFi acquired resistance in melanoma with impaired PTEN. We showed that inhibition of PERK by shRNAs or a small molecular inhibitor enhanced the sensitivity of resistant inactivated PTEN melanoma cells to BRAFi and blocked their growth in vitro and in vivo, suggesting that PERK is a new prognostic marker and therapeutic target for BRAFi-resistant BRAF-mutant melanoma with impaired PTEN. Our findings, along with our previous study demonstrating AXL/AKT axis-mediated resistance to BRAFi in melanoma with wild-type PTEN^26^, have uncovered a mechanism by which PTEN status contributes to acquired resistance to BRAFi, and offers a rational strategy to guide clinical testing in pre-identified subsets of patients who relapse during treatment with BRAFi.

## Methods

### Plasmids, antibodies, cell lines, cell culture, and reagents

Plasmids: PERK shRNAs expressing plasmids were purchased from Open Biosystems (GE Dharmacon, Lafayette, CO). PTEN specific CRISPR/cas9 knockout vector (cat# sc-400103) and the specific PTEN homology-directed repair PTEN–HDR vector (cat# sc-400103-HDR) for humans were purchased from Santa Cruz (Dallas, TX) directedly to knockout the PTEN gene specifically. PTEN expressing plasmid, PERK wild-type, and PERK mutant expressing plasmids were provided by Addgene (Cambridge, MA). Antibodies: anti-Bip/grp78 (1:1000, cat# 3177), anti-IRE1α (1:1000, cat# 3294), anti-eIF2α (1:1000, cat# 5324), anti-p-eIF2α (1:1000, cat# 3398), anti-PERK (1:1000, cat# 3192), anti-p-PERK (1:1000, cat# 3179), anti-Calenexin (1:1000, cat# 2679), anti-tubulin (1:1000, cat# 2146), anti-PTEN (1:1000, cat# 9188), anti-GAPDH (1:1000, cat# 5174), anti-CHOP (1:1000, cat# 2895), anti-Atf4 (1:1000, cat# 11815), anti-Atf6 (1:1000, cat#65880), anti-cleaved Caspase-3 (Asp175) (1:1000, cat# 9664), anti-Caspase-3 (1:1000, cat# 9662), anti-PARP (1:1000, cat# 9532), anti-cleaved PARP (Asp214) (1:1000, cat #5625), anti-Caspase-9 (1:1000, cat# 9502), anti-cleaved Caspase-9 (Asp330) (1:1000, cat# 7237), anti-Caspase-7 (1:1000, cat# 12827), anti-cleaved Caspase-7 (Asp198) (1:1000, cat# 9491), anti-β actin (1:1000, cat# 4970), anti-Akt (1:1000, cat# 4685), anti-p-Akt (1:1000, cat# 4060), anti-Erk1/2 (1:1000, cat# 4695), and anit-p-Erk1/2 (1:1000, cat# 4370) were purchased from Cell Signaling (Danvers, MA, USA); anti-phospho-PERK (1:1000, cat# PA5-40294) was purchased from Thermo Fisher Scientific (Waltham, MA USA); anti-β-actin antibody (1:500, sc69879) was purchased from Santa Cruz (Dallas, TX). Human melanoma cell lines: A375sm was a gift from Dr. Isaiah Fidler (M.D. Anderson Medical Center, Houston, TX); WM88, WLH6215 cell lines were a gift from Dr. Meenhard Herlyn (The Wistar Institute, Philadelphia, PA); Sk-mel 28 was obtained from American Type Culture Collection (ATCC, Manassas, VA). The BRAFi-resistant cell lines (A375smR, WM88R, WLH6215R, and Sk-mel28R) were established in our previous study^26^. The stable expressing cell lines were established by transfection using lipofectamine 2000 reagent (Invitrogen, Carlsbad, CA) and selected by antibiotics G418 or puromycin (Sigma, St. Louis, MO). All cell lines were tested negative with mycoplasma and authenticated using short tandem repeat (STR) DNA profiling routinely. The cell lines for in vivo animal studies were negative for infectious microbes by molecular testing of biological materials (MTBM) test [the animal proposal LCBG023, approval by NCI-Bethesda Animal Care and Use Committee (ACUC)]. A BRAF inhibitor (PLX4032) and a PERK inhibitor (GSK2606414) were purchased from Selleckchem (Houston, TX) or MCE (MedChemExpress, Monmouth, NJ).

### PTEN phosphatase activity assay

Detection and Quantification of PTEN Phosphatase Activity were measured by PTEN Activity ELISA assay described in the PTEN Activity ELISA kit (K-4700, Echelon Biosciences Inc, Utah, USA). Briefly, Transfer 200 μg of the cell lysate to a fresh, cold, 1.5 mL centrifuge tube. Add 8 μL of the anti-PTEN antibody (Cell Signaling) to the lysate. Incubate overnight at 4 °C with agitation. Add 60 μL of the 50% Protein A agarose beads to the mixture and incubate for 2 h at 4 °C. Briefly centrifuge to pellet beads. Discard the supernatant. Wash the bead complex three times with ice-cold PBS. Centrifuge and discard the solution after each wash. Wash once with PTEN Reaction Buffer. Resuspend bead complex in 30 μL of PTEN Reaction buffer. Proceed immediately with the PTEN reactions by adding 30 μL of the 16 μM PI(3,4,5)P3 Substrate to the bead complex. The product PIP2 of the PTEN enzyme reaction was measured.

### Establishment of PLX4032-resistant melanoma cells^26^

Aliquots of melanoma cells in the exponential growth phase were seeded into 75 cm^2^ cell culture flasks. PLX4032 (1 μmol/mL) was added for 48 h during the mitotic phase. Then the cells were transferred into a drug-free culture medium until the next mitotic phase (around 7–10 d), after which PLX4032 was added for the next 48 h at twice the previous concentration. We continued this process in a stepwise increasing concentration of PLX4032 while observing cell death every day, changing to the fresh complete culture medium, and regularly performing the CCK8 assay. This process was continued for 6 months until the melanoma cells grew stably in the PLX4032-containing medium.

### Cellular proliferation and drug treatments

Cell proliferation experiments were carried out in 96-well plates and measured using CCK8 assay^26,57,59^. Cells in the exponential growth phase were inoculated into each well at a density of 3 × 10^3^ cells per well, with five wells for each set of conditions. Drug treatments were initiated at 24 h and lasted for 72 h. After then, 10 μL CCK8 was added to each well, and the cells were incubated at 37 °C under 5% CO2 for 4 h. The optical density (OD) of each well at a wavelength of 450 nm was determined using a microplate reader. Cell viability was calculated according to the following equation: (drug-supplemented OD-blank control OD)/ (normal control OD-blank control OD) × 100%. Origin 6.1 or GraphPad prism 8 software was utilized to plot the survival versus drug concentration curve and calculate the 50% inhibitory concentration (IC50). The resistance index (RI) was calculated as the ratio between the IC50 value of resistant cells and parental cells.

### Western blot analysis

Immunoblots were performed on lysates generated from cultured cells and tissues solubilized in RIPA buffer^26,57,59^. All blots derive from the same experiment and they were processed in parallel.

### Preclinical drug treatments

Parents and resistant melanoma cell lines Sk-mel28 and Sk-mel28R or WLH6215 and WLH6215R cells (200 μL, 1 × 10e6) were transplanted into athymic nude or NSG male mice (purchased from The Jackson Laboratory) between 4 and 6 weeks of age subcutaneously (SQ). Mice harboring the inoculated tumor cells 14 days (Sk-mel28 and Sk-mel28R) or 17 days (WLH6215 and WLH6215R) after transplantation with similar tumor size were randomly assigned to different treatment groups using a parallel-group design and treated with either 50 or 75 mg/kg of the PLX4032 twice daily or 7.5 mg/kg (intratumoral) or 150 mg/kg of PERK inhibitor GSK2606414 once daily by oral gavage for 22 days. The cage of the mouse and treatments were coded with a number. The tumor size was monitored by measuring the length (*L*) and width (*W*) using a caliper every 2 days, and the volumes were calculated via the formula: (*L* × *W*^2^) × 0.5. Each group was initialed with 10 mice [based on the typical power of 80 to 90%, 6 animals is considered adequate for hypothesis testing]^26,57,59^.

### Evaluation of interactions between BRAF inhibitor and PERK inhibitor

Checkerboard mothed: BRAFi-resistant melanoma Sk-mel28R and WLH6215R cells were treated with PLX4032 and GSK2606414 in an 8 × 8 concentration grid (checkerboard design) for 72 h. Cell viability was determined by CCK8. The experimental data were analyzed independently with the two synergy models (Bliss and Loewe) using the Combenefit software^60^. A constant-ratio experimental design: BRAFi-resistant melanoma Sk-mel28R and WLH6215R cells were treated with PLX4032, GSK2606414, or a combination of PLX4032 and GSK2606414 at a constant concentration ratio of 2:1 for 72 h. Cell viability was determined by CCK8. The experimental data were analyzed using the CompuSyn software^61,62^. According to the Chou and Talalay mathematical model for drug interactions, the combination index (CI) was calculated for cells receiving combination therapy. The resulting CI theorem of Chou–Talalay offers a quantitative definition for an additive effect (CI = 1), synergism (CI < 1), and antagonism (CI > 1) in drug combinations^62^.

### Ethics

All mouse procedures were performed according to National Institutes of Health guidelines. The animal studies were under the animal proposal LCBG023, approved by the National Cancer Institute-Bethesda Animal Care and Use Committee (ACUC) in the United States of America.

### Statistical analysis

All data were represented as the mean of at least triplicate samples ± standard deviation. All statistical analyses in this study were performed using SPSS 13.0 and GraphPad Prism 8. A two-tailed Student’s *t*-test or one-way ANOVA was used to test the differences in sample means for data with normally distributed means. The significance of mean values between two groups was analyzed by Student’s *t*-test or multiple *t*-tests. *p*-values <0.05 were considered statistically significant.

### Reporting summary

Further information on research design is available in the Nature Research Reporting Summary linked to this article.

## Supplementary information

Supplementary Information

Reporting Summary

## Data Availability

Data are available from the corresponding author upon reasonable request.
