# Supplementary material for: PERK mediates resistance to BRAF inhibition in melanoma with impaired PTEN
Source: NPJ Precis Oncol. 2021 Jul 19;5:68. doi: 10.1038/s41698-021-00207-x (PMC8289936; doi:10.1038/s41698-021-00207-x)
Supplement: Supplementary file 1 — Supplementary Information [file 41698_2021_207_MOESM1_ESM.pdf]

Supplementary information

PERK shRNAs expressing plasmids were purchased from Open Biosystems (GE Dharmacon, Lafayette, CO). The clone id and sequences of mature antisense are:

1. V3SH7590-226723129, Clone Id: V3SVHSHC_6660779, Mature Antisense: AACACTGAAATTCCACTTC
2. V3SH7590-226355776, Clone Id: V3SVHSHC_6293423, Mature Antisense: TATAGCTTCAGGTCGTTCC
3. V3SH7590-229279045, Clone Id: V3SVHSHC_9216695, Mature Antisense: CAGTCATCTACTTTGTTTT

Supplementary figures

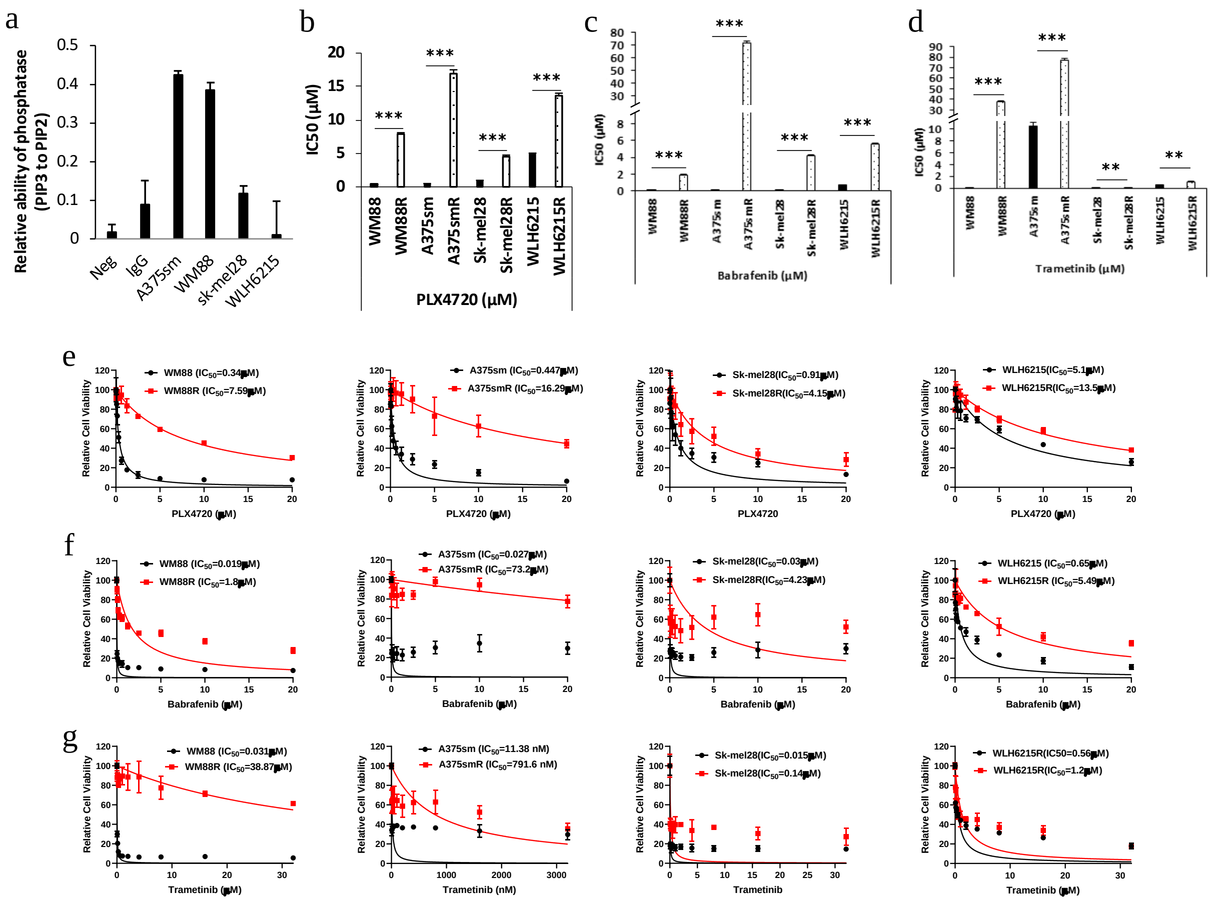

Supplementary Figure 1. (A) PTEN phosphatase activity in the melanoma cell lines. Detection and Quantification of PTEN Phosphatase Activity were measured by PTEN Activity ELISA assay described in the PTEN Activity ELISA kit (K-4700, Echelon Biosciences Inc, Utah, USA). PTEN proteins were pull down from cell lysate of melanoma cells by PTEN antibody. Neg,

negative control; IgG, IgG control; (B) (C) and (D) The values of 50%-inhibitory-concentration (IC50) of 4 pairs parental (WM88, A375sm, Sk-mel28 and WLH6215) and resistant (WM88R, A375smR, Sk-mel28R, and WLH6215R) human melanoma cells were determined using CCK8 assays. Error bars denote s.d. for biological three repeats. Results are statistically significant between parental and resistant groups by Student's t-test (* p<0.05, **p<0.01, *** p<0.001). (E) (F) and (G)The cell viability curves of 4 pairs of parental (WM88, A375sm, Sk-mel28, and WLH6215) and resistant (WM88R, A375smR, Sk-mel28R, and WLH6215R) human melanoma cells were determined using CCK8 assays. Error bars denote s.d. for biological three repeats.

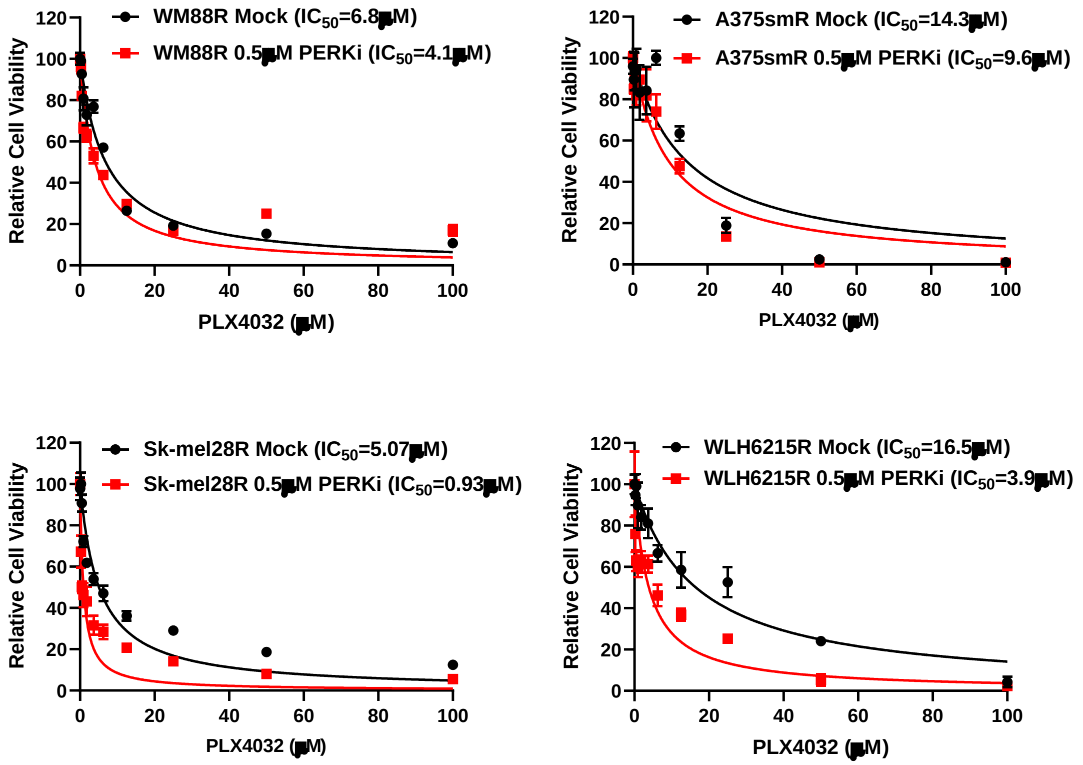

Supplementary Figure 2. The cell viability curves of 4 pairs of parental (WM88, A375sm, Sk-mel28, and WLH6215) and resistant (WM88R, A375smR, Sk-mel28R, and WLH6215R) human melanoma cells treated with the combination of PLX4032 and PERKi GSK2606414 were determined using CCK8 assays. Mock, DMSO as control; PERKi, PERK inhibitor GSK2606414. Error bars denote s.d. for biological three repeats.

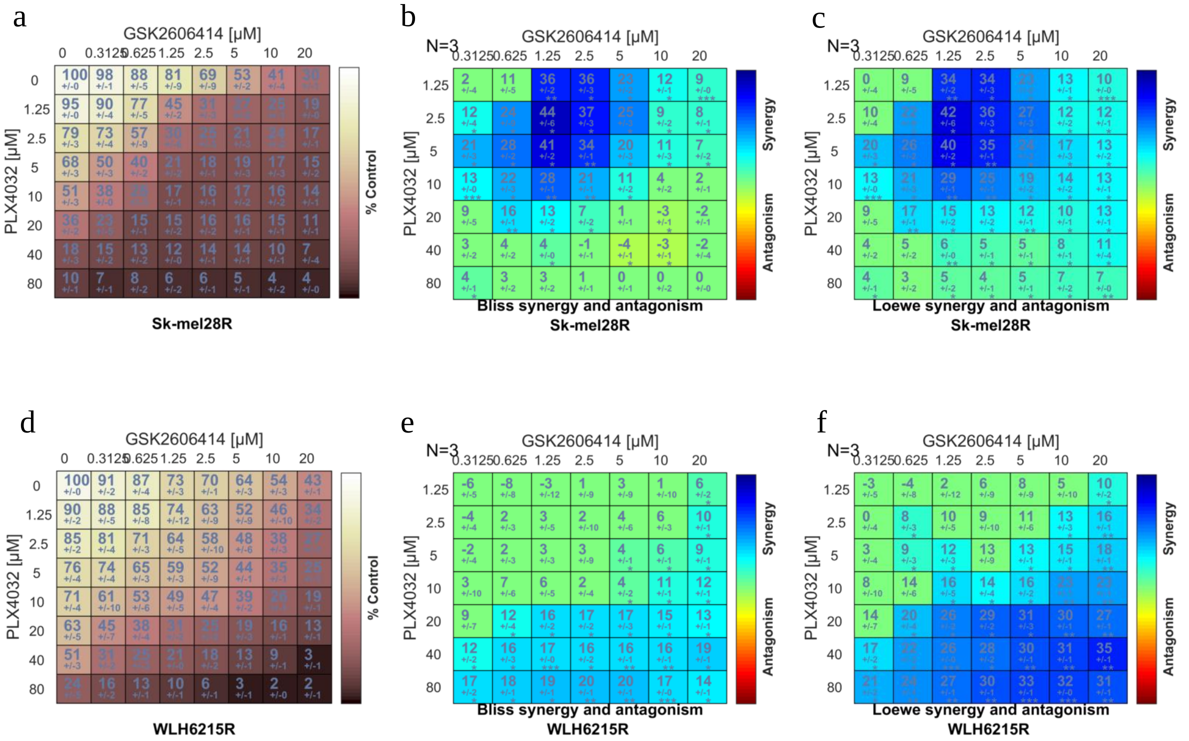

Supplementary Figure 3. A checkerboard method to evaluate interactions between BRAF inhibitor PLX4032 and PERK inhibitor GSK2606414. BRAFi resistant melanoma Sk-mel28R and WLH6215R cells were treated with PLX4032 and GSK2606414 in 8 x 8 concentration grid (checkerboard design) for 72 hours. Cell viability was determined by CCK8. The experimental data were analyzed independently with the two synergy models [Bliss (B, E) and Loewe (C, F)] using the Combenefit software. (A) and (D) Values are percentage growth inhibition compared with control. Data, mean ± SD; n = 3.

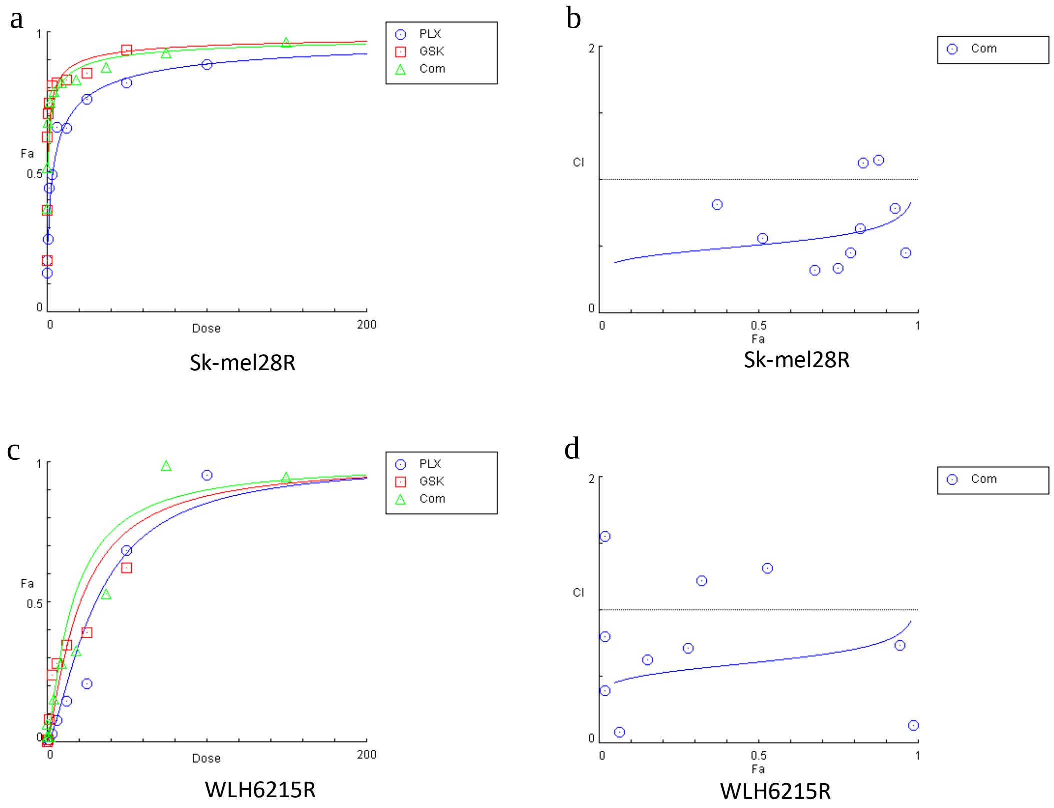

Supplementary Figure 4. A constant -ratio experimental design to evaluate interactions between BRAF inhibitor PLX4032 and PERK inhibitor GSK2606414. BRAFi resistant melanoma Sk-mel28R and WLH6215R cells were treated with PLX4032, GSK2606414, or a combination of PLX4032 and GSK2606414 at a constant concentration ratio of 2:1 for 72 hours. Cell viability was determined by CCK8. The experimental data were analyzed using the CompuSyn software. (A) and (C) Graphs of dose-effect of PLX4032 (PLX), GSK2606414 (GSK) and combination (com); (B) and (D) Graphs of combination indexes (CI).

Figure 1a

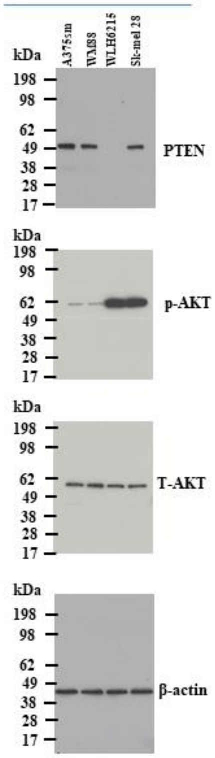

Figure 1c

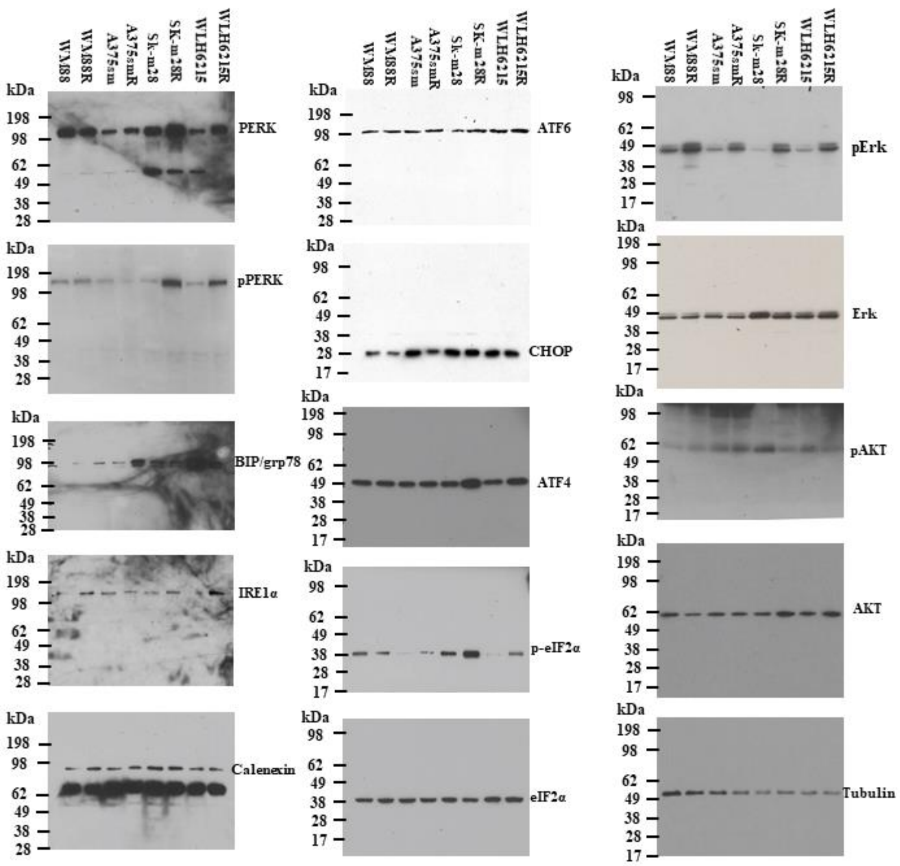

Figure 3a

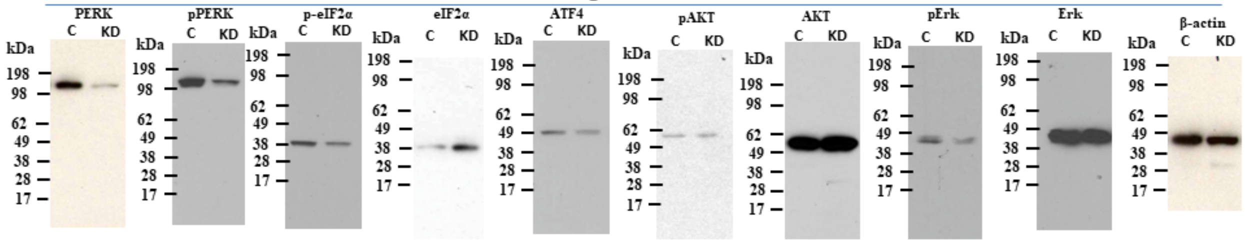

Figure 3c

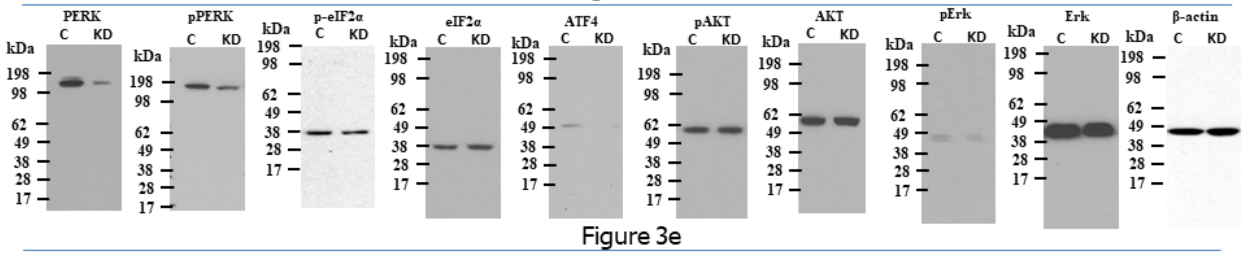

Figure 3e

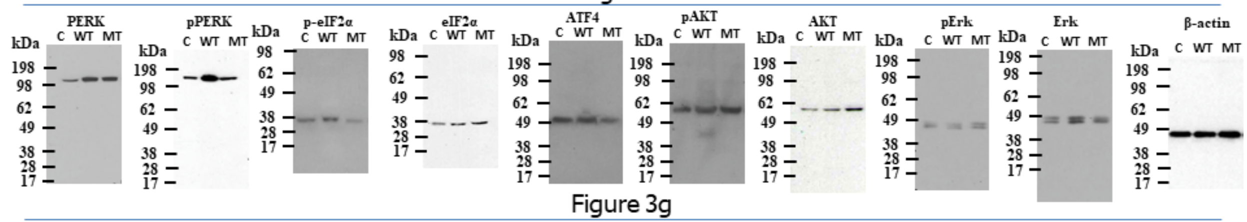

Figure 3g

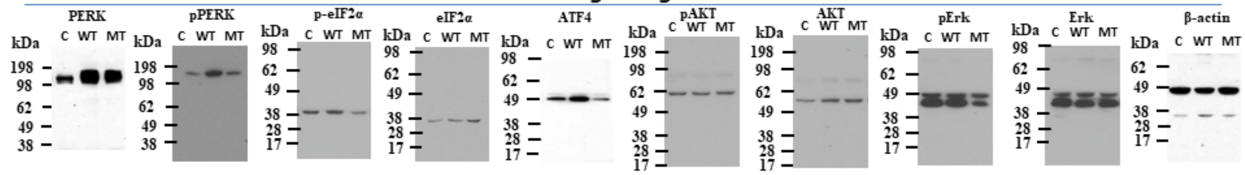

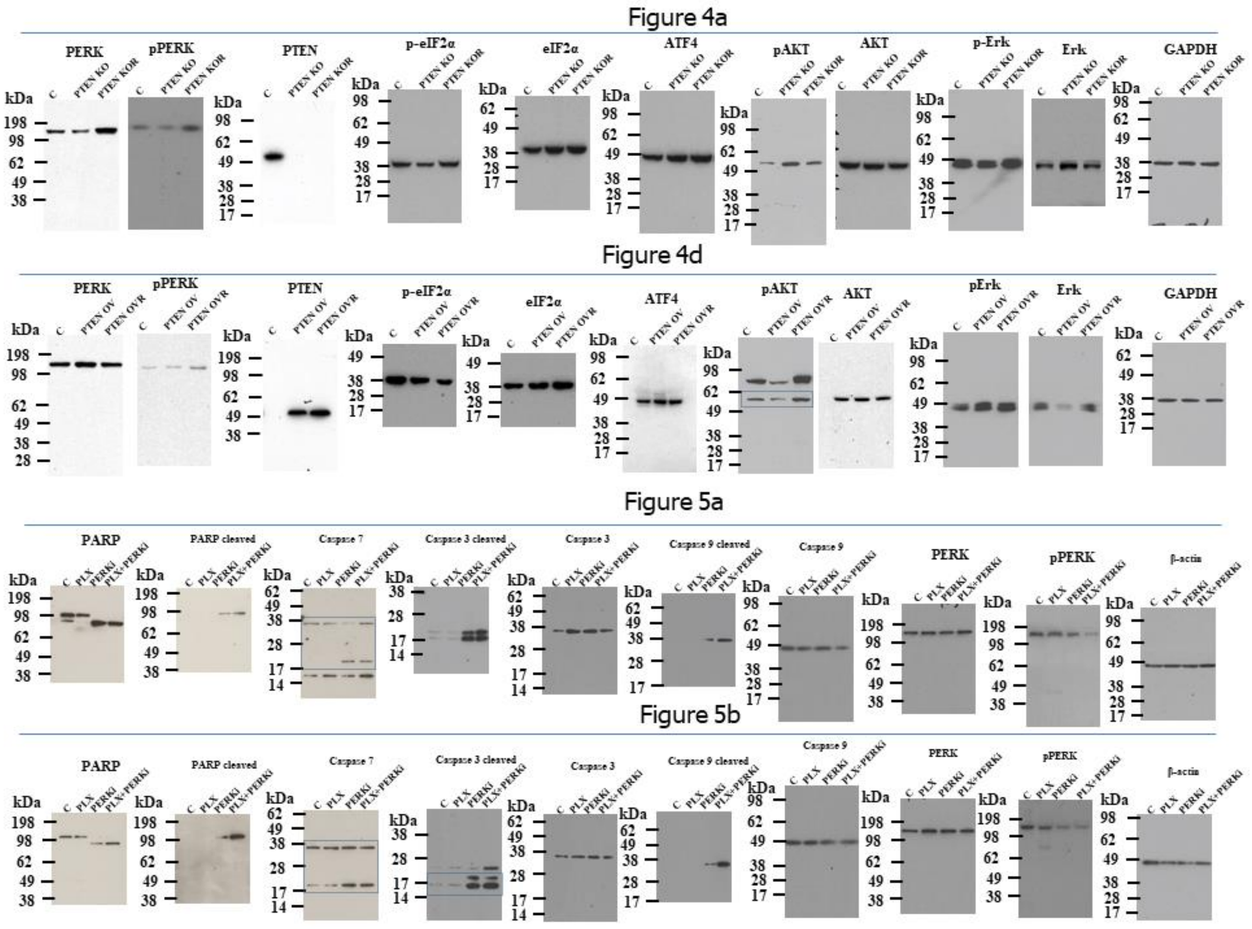

Supplementary Figure 5 un-cropped images.
