# Supplementary material for: PERK mediates resistance to BRAF inhibition in melanoma with impaired PTEN
Source: NPJ Precis Oncol. 2021 Jul 19;5:68. doi: 10.1038/s41698-021-00207-x (PMC8289936; doi:10.1038/s41698-021-00207-x)
Supplement: Supplementary file 2 — Reporting Summary [file 41698_2021_207_MOESM2_ESM.pdf]

nature research

Corresponding author(s): Yanlin Yu

Reporting Summary

Nature Research wishes to improve the reproducibility of the work that we publish. This form provides structure for consistency and transparency in reporting. For further information on Nature Research policies, see Authors & Referees and the Editorial Policy Checklist.

Statistical parameters

When statistical analyses are reported, confirm that the following items are present in the relevant location (e.g. figure legend, table legend, main text, or Methods section).

n/a	Confirmed	
☐	☒	The exact sample size (n) for each experimental group/condition, given as a discrete number and unit of measurement
☐	☒	An indication of whether measurements were taken from distinct samples or whether the same sample was measured repeatedly
☐	☒	The statistical test(s) used AND whether they are one- or two-sided<br>*Only common tests should be described solely by name; describe more complex techniques in the Methods section.*
☐	☒	A description of all covariates tested
☐	☒	A description of any assumptions or corrections, such as tests of normality and adjustment for multiple comparisons
☐	☒	A full description of the statistics including central tendency (e.g. means) or other basic estimates (e.g. regression coefficient) AND variation (e.g. standard deviation) or associated estimates of uncertainty (e.g. confidence intervals)
☐	☒	For null hypothesis testing, the test statistic (e.g. F, t, r) with confidence intervals, effect sizes, degrees of freedom and P value noted<br>*Give P values as exact values whenever suitable.*
☒	☐	For Bayesian analysis, information on the choice of priors and Markov chain Monte Carlo settings
☒	☐	For hierarchical and complex designs, identification of the appropriate level for tests and full reporting of outcomes
☒	☐	Estimates of effect sizes (e.g. Cohen's d, Pearson's r), indicating how they were calculated
☐	☒	Clearly defined error bars<br>*State explicitly what error bars represent (e.g. SD, SE, CI)*

Our web collection on statistics for biologists may be useful.

Software and code

Policy information about availability of computer code

Data collection	microplate reader for CCK8 assay
Data analysis	Microsoft Excel and GraphPad Prism 8, student T test

For manuscripts utilizing custom algorithms or software that are central to the research but not yet described in published literature, software must be made available to editors/reviewers upon request. We strongly encourage code deposition in a community repository (e.g. GitHub). See the Nature Research guidelines for submitting code & software for further information.

Data

Policy information about availability of data

All manuscripts must include a data availability statement. This statement should provide the following information, where applicable:

- Accession codes, unique identifiers, or web links for publicly available datasets
- A list of figures that have associated raw data
- A description of any restrictions on data availability

The data that support the findings of this study are available form the corresponding author upon reasonable request.

Field-specific reporting

Please select the best fit for your research. If you are not sure, read the appropriate sections before making your selection.

☒ Life sciences ☐ Behavioural & social sciences

For a reference copy of the document with all sections, see nature.com/authors/policies/ReportingSummary-flat.pdf

Life sciences

Study design

All studies must disclose on these points even when the disclosure is negative.

Sample size	all cell viability were done with 3 biological three repeats each with 3 or 4 technical replication. Animal experiments were carried out based on previous power analysis and publications. Xenograft models from group's tumor size was measured for the length (L) and width (W) using caliper. The sample sizes of 8 or greater animals based on expected standard deviation for the sample t-test.
Data exclusions	*Describe any data exclusions. If no data were excluded from the analyses, state so OR if data were excluded, describe the exclusions and the rationale behind them, indicating whether exclusion criteria were pre-established.*
Replication	all cell viability were done with 3 biological three repeats each with 3 or 4 technical replication.
Randomization	In animal experiments , animals were randomized to injection and treatment groups.
Blinding	In animal experiments, treatments and measurement of tumor growth were carried out by animal technician.

Materials & experimental systems

Policy information about availability of materials

n/a	Involved in the study
☐	☒ Unique materials
☐	☒ Antibodies
☐	☒ Eukaryotic cell lines
☐	☒ Research animals
☒	☐ Human research participants

Unique materials

Obtaining unique materials	BRAFi resistant melanoma cell lines were generated by our group.

Antibodies

Antibodies used	Cell Signaling Technology (CST) Antibodies: anti-Bip/grp78, cat#3177; anti-IRE1α, cat#3294; anti-eIF2α, cat# 5324; anti-p-eIF2α cat# 3398; anti-PERK, cat#3192; anti-p-PERK, cat#3179; anti-Calenexin, cat#2679; anti-tubulin, cat# 2146; anti-PTEN, cat#9188; anti-GAPDH, cat#5174; anti-CHOP, cat#2895; anti-Atf4, cat#11815; anti-Atf6, cat#65880; anti-cleaved Caspase -3(Asp175), cat#9664; anti-Caspase-3, cat#9662; anti-PARP, cat#9532; anti-cleaved PARP (Asp214), cat#5625; anti-Caspase-9, cat9502; anti-cleaved Caspase-9 (Asp330), cat#7237; anti-Caspase-7, cat#12827; and anti-cleaved Caspase-7 (Asp198), cat#9491; anti-β actin, cat#4970; anti-Akt, cat#4685; anti-p-Akt, cat#4060; anti-Erk1/2, cat# 4695 and anit-p-Erk1/2, cat#4370.
Validation	All antibodies purchased from Cell Signaling (Danvers, MA, USA) weew QC tessted and validated in human cells for western blot.

Eukaryotic cell lines

Policy information about cell lines

Cell line source(s)	A375sm was a gift from Dr. Isaiah Fidler (M.D. Anderson Medical Center, Houston, TX); WM88, WLH6215 cell lines were a gift from Dr. Meenhard Herlyn (The Wistar Institute, Philadelphia, PA); Sk-mel 28 was obtained from American Type Culture Collection (ATCC, Manassas, VA).
Authentication	Short-tandem repeat profiling of cell lines were used to confirm the identity of the cells.
Mycoplasma contamination	No mycoplasma contamination. All cell lines were also tested by health diognostic laboratory in NIH

Commonly misidentified lines
(See ICLAC register)

N/A

Research animals

Policy information about studies involving animals; ARRIVE guidelines recommended for reporting animal research

Animals/animal-derived materials

n/a

Method-specific reporting

n/a	Involved in the study
☒	☐ ChIP-seq
☒	☐ Flow cytometry
☒	☐ Magnetic resonance imaging
